# Ma Huang Tang Suppresses the Production and Expression of Inflammatory Chemokines via Downregulating STAT1 Phosphorylation in HaCaT Keratinocytes

**DOI:** 10.1155/2016/7831291

**Published:** 2016-10-26

**Authors:** Hye-Sun Lim, Chang-Seob Seo, Seong-Eun Jin, Sae-Rom Yoo, Mee-Young Lee, Hyeun-Kyoo Shin, Soo-Jin Jeong

**Affiliations:** ^1^Herbal Medicine Research Division, Korea Institute of Oriental Medicine, Daejeon 34054, Republic of Korea; ^2^K-herb Research Center, Korea Institute of Oriental Medicine, Daejeon 34054, Republic of Korea; ^3^Korean Medicine Life Science, University of Science & Technology, Daejeon 34113, Republic of Korea

## Abstract

Ma huang tang (MHT) is a traditional herbal medicine comprising six medicinal herbs and is used to treat influenza-like illness. However, the effects of MHT on inflammatory skin diseases have not been verified scientifically. We investigated determining the inhibitory effects of MHT against inflammation responses in skin using HaCaT human keratinocyte cells. We found that MHT suppressed production of thymus and activation-regulated chemokine (TARC/CCL17), macrophage-derived chemokine (MDC/CCL22), regulated on activation of normal T-cell expressed and secreted (RANTES/CCL5), and interleukin-8 (IL-8) in tumor necrosis factor-*α* (TNF-*α*) and interferon-*γ*- (IFN-*γ*-) stimulated HaCaT cells. Consistently, MHT suppressed the mRNA expression of TARC, MDC, RANTES, and IL-8 in TNF-*α* and IFN-*γ*-stimulated cells. Additionally, MHT inhibited TNF-*α* and IFN-*γ*-stimulated signal transducer and activator of transcription 1 (STAT1) phosphorylation in a dose-dependent manner and nuclear translocation in HaCaT cells. Our finding indicates that MHT inhibits production and expression of inflammatory chemokines in the stimulated keratinocytes by downregulating STAT1 phosphorylation, suggesting that MHT may be a possible therapeutic agent for inflammatory skin diseases.

## 1. Introduction

Atopic dermatitis (AD) is a frequently occurring inflammatory skin disease associated with severe itching and allergies to environmental factors [1]. AD is characterized by the predominant infiltration of macrophages, mast cells, eosinophils, and other inflammatory cells and increased secretion of Th2-related response factors by the production of tumor necrosis factor-*α* (TNF-*α*) and interferon-*γ* (IFN-*γ*) [2].

Keratinocytes play a pivotal role in the development of inflammatory skin diseases including AD. The cells produced different chemokines and cytokines, especially thymus and activation-regulated chemokine (TARC/CCL17), macrophage-derived chemokine (MDC/CCL22), regulated on activation of normal T-cell expressed and secreted (RANTES/CCL5), and interleukin-8 (IL-8) in response to stimulation by TNF-*α*/IFN-*γ* [3, 4]. These mediators are thought to be crucial regulators of the pathogenesis of AD. Additionally, the stimulation of keratinocytes by TNF-*α*/IFN-*γ* leads to the activation of various signaling pathways that involve caspases, mitogen activated protein kinases, nuclear factor-kappa B, and signal transducer and activator of transcription 1 (STAT1) which subsequently increase the expression of inflammatory mediators [5].

Ma huang tang (MHT) comprises six herbal medicines and has traditionally been used in the treatment of sweating, asthma, and febrile disease, such as influenza-like illness (high fever, headache, and cough) [6, 7]. Previous research reported that MHT has antipyretic effect in pediatric patients [8]. Other studies showed that MHT has antiasthmatic effects [9]. However, there has been no investigation focusing on the skin inflammatory effects of MHT. Therefore, we investigated the effects and action mechanisms of MHT on the inflammatory mediators using HaCaT human keratinocytes.

## 2. Materials and Methods

### 2.1. Plant Materials

MHT consisting of 6 herbs (Table 1), Ephedrae Herba, Cinnamomi Ramulus, Glycyrrhizae Radix et Rhizoma, Armeniacae Semen, Zingiberis Rhizoma Crudus, and Allii Radix, was purchased from Kwangmyungdang Medicinal Herbs (Ulsan, Korea). All raw herbal medicines were confirmed by pharmacognosists, Professor Je-Hyun Lee (College of Oriental Medicine, Dongguk University, Gyeongju, Republic of Korea) and Professor Young-Bae Seo (College of Oriental Medicine, Daejeon University, Gyeongju, Republic of Korea). A voucher specimen (2012–KE47–1~KE47–6) has been deposited at the K-herb Research Center, Korea Institute of Oriental Medicine (KIOM).

### 2.2. Chemicals and Reagents

Cinnamaldehyde (≥98.0%), cinnamic acid (≥98.0%), liquiritin (≥99.0%), 6-gingerol (≥98.0%), and glycyrrhizin (≥99.0%) were purchased from Wako (Osaka, Japan). Amygdalin and coumarin (both ≥99.0%) were purchased from Sigma-Aldrich (St. Louis, MO, USA). Liquiritin apioside (≥98.0%) was purchased from Shanghai Sunny Biotech (Shanghai, China) and ephedrine HCl (≥95.0%) was provided from Ministry of Food and Drug Safety. High-performance liquid chromatography (HPLC) grade, methanol, acetonitrile, and water were purchased from J. T. Baker (Phillipsburg, NJ, USA). Reagent grade, trifluoroacetic acid was purchased from Sigma-Aldrich (St. Louis, MO, USA).

### 2.3. Preparation of MHT Water Extract

MHT water extract was prepared in our laboratory, KIOM. Namely, the 6 crude herbs, Ephedrae Herba (1,744 g), Cinnamomi Ramulus (1,163 g), Glycyrrhizae Radix et Rhizoma (349 g), Armeniacae Semen (581 g), Zingiberis Rhizoma Crudus (581 g), and Allii Radix (581 g), were mixed and extracted with 50 L of distilled water at 100°C for 2 h under pressure (98 kPa) using an electric extractor (COSMOS-660; Kyungseo Machine Co., Incheon, Korea). The extracted solution was filtrated through the standard sieve (number 270, 53 *μ*m; Chung Gye Sang Gong Sa, Seoul, Korea) and then the filtered solution was freeze-dried to obtain a powder using freezing dryer, PVTFD10RS (IlShinBioBase, Yangju, Korea). The amount of freezing-dried MHT powder obtained was 226.2 g (yield: 4.5%).

### 2.4. Chromatographic Analysis of MHT Sample

Chromatographic analysis of the marker components in MHT was performed using the Shimadzu Prominence LC-20A series HPLC system (Kyoto, Japan) equipped with photodiode array (PDA) detector and Lab Solution software (Version 5.54 SP3, Shimadzu, Kyoto, Japan). Waters SunFire C_18_ analytical column (250 × 4.6 mm; 5 *μ*m, Milford, MA, USA) was used for the separation of the main components as the stationary phase and maintained at 40°C. The mobile phases consisted of 0.1% (v/v) trifluoroacetic acid in distilled water (A) and acetonitrile (B) and the gradient elution for chromatographic separation was as follows: 10–60% B for 0–30 min, 60–100% B for 30–40 min, 100% B for 40–45 min, 100–10% B for 45–50 min, and 10% B for 50–60 min. The flow rate was 1.0 mL/min and injection volume was 10 *μ*L.

### 2.5. Cell Culture

The HaCaT human keratinocyte cell line was obtained from CLS Cell Lines Service GmbH (Eppelheim, Baden-Württemberg, Germany). The HaCaT cells were cultured in Dulbecco's modified Eagle's medium (DMEM, Gibco-BRL, Invitrogen Life Technologies, Inc., Carlsbad, CA, USA) supplemented with 10% heat-inactivated fetal bovine serum (FBS, Gibco-BRL), penicillin (100 *μ*g/mL; Gibco-BRL), and streptomycin (100 *μ*g/mL; Gibco-BRL) in an incubator containing 5% CO_2_ at 37°C.

### 2.6. Cytotoxicity Assay

Cell viability was assessed using a Cell Counting Kit-8 assay (CCK-8 from Dojindo, Kumamoto, Japan) according to the manufacturer's instructions. HaCaT cells (1 × 10^3^ cells/well) were incubated in 96-well plates with various concentrations of the MHT for 24 h. CCK-8 reagent was added to each well and cells were incubated for an additional 4 h. The absorbance was measured at 450 nm using a Benchmark Plus microplate reader (Bio-Rad Laboratories, Hercules, CA, USA). The percentage of viable cells was calculated as follows: cell viability (%): (mean absorbance in test well/mean absorbance in control well) × 100.

### 2.7. Measurement of Chemokine Production

HaCaT cells (1 × 10^6^ cells/well) were cultured in 6-well plates. After reaching confluency, the cells were washed and treated with MHT in 1 mL of serum-free medium containing TNF-*α* and IFN-*γ* (TI, each 10 ng/mL; R&D Systems Inc., Minneapolis, MN, USA) for 24 h. The supernatant fractions were harvested, and production of TARC, MDC, RANTES, and IL-8 was determined using a sandwich immunoassay, performed according to the protocols provided by R&D Systems.

### 2.8. Reverse Transcription-Polymerase Chain Reaction (RT-PCR)

Total RNA was isolated using TRIzol reagent according to the manufacturer's instructions (Invitrogen Life Technologies, Inc.). HaCaT cells (1 × 10^6^ cells/well) were cultured to 80–90% confluency in 6-well plates. When the cells reached confluence, the cells were washed and treated with MHT in 1 mL serum-free medium (Gibco-BRL) supplemented with TI for 24 h. Silymarin (Sigma-Aldrich Inc., St. Louis, MO) was used as a positive control drug. Total RNA (1 *μ*g/mL) was then converted into cDNA using an iScript cDNA Synthesis Kit (Bio-Rad Laboratories, Inc.), containing oligo-dT primers. Diethylpyrocarbonate-treated water was added to a final volume 20 *μ*L followed by incubation at 42°C for 30 min using a Bio-Rad iCycler apparatus (Bio-Rad Laboratories, Inc.). For PCR amplification, gene-specific primers sequences are listed in the 5′ to 3′ orientation in Table 2. The PCR reaction mixture contained 1 *μ*L cDNA and 1.56 *μ*L *γ*Taq PCR master mix (EBT-1014; Elpis Biotech, Inc., Daejeon, Korea), which contained 1.5 mM MgCl_2_, 0.1 *μ*M of each forward and reverse primer and 7.44 *μ*L water in a final volume of 10 *μ*L. The thermocycling program comprised initial denaturation at 94°C for 5 min, followed by 25 cycles of denaturation at 94°C for 30 sec, annealing at 64°C for 1 min, extension at 72°C for 1 min 30 sec for all of the chemokines, and 25 cycles of denaturation at 94°C for 30 sec, annealing at 52°C for 1 min, and extension at 72°C for 1 min 30 sec for GAPDH. A final extension step was conducted at 72°C for 7 min. The amplified products were separated by 1.5% agarose gel and visualized using Loading STAR staining (A750; DYNE Bio, Seongnam, Korea). The relative expression levels of TARC, MDC, RANTES, and IL-8 mRNA were normalized to those of GAPDH mRNA using a Chemi-Doc Band Analysis system (Bio-Rad Laboratories, Inc.).

### 2.9. Western Blotting

HaCaT cells were treated with various concentrations of MHT for 1 h and then incubated in the presence of TI for 30 min. The cells were collected by centrifugation, washed twice with PBS, and suspended in the NE-PER Nuclear and Cytoplasmic Extraction Reagents (Thermo Scientific, Rockford, IL) containing protease inhibitors. The protein concentration was determined using a protein assay reagent (Bio-Rad Laboratories, Inc.) according to the manufacturer's instructions. Nuclear protein (30 *μ*g) was resolved by 10% sodium dodecyl sulfate-polyacrylamide gel electrophoresis (SDS-PAGE) and transferred to a polyvinylidene difluoride membrane. The membrane was incubated with blocking solution [5% skim milk in Tris-buffered saline containing 0.1% Tween 20 (TBST)], followed by an overnight incubation at 4°C with the appropriate primary antibody. The following primary antibodies and dilution were used: anti-STAT1 and antiphospho-STAT1 (1 : 1000 dilution; Abcam, Cambridge, UK). The membranes were washed three times with TBST and then incubated with a 1 : 3000 dilution of a horseradish peroxidase- (HRP-) conjugated secondary antibody (Jackson ImmunoResearch, PA) for 1 h at room temperature. The membranes were again washed three times with TBST and then developed using an enhanced chemiluminescence kit (Thermo scientific, Rockford, IL). Image capture was performed using Chemi-Doc XRS^+^ system (Bio-Rad Laboratories, Inc.).

### 2.10. Immunofluorescence Staining

HaCaT cells were seeded onto glass coverslips and incubated with TI in the absence or presence of MHT (500 *μ*g/mL) for 30 min. The cells were fixed in 4% paraformaldehyde and 100% acetone, blocked in 0.5% bovine serum albumin, and incubated with anti-STAT1 antibody (Cell Signaling, Danvers, MA) for 1 h at room temperature. Then, fluorescein isothiocyanate-conjugated anti-rabbit immunoglobulin G antibody (Invitrogen, Carlsbad, CA, USA) was used as a secondary antibody. The immunostained cells were mounted with medium containing 4′6-diamidino-2-phenylindole (DAPI) and visualized using an Olympus FLUOVIEW FV10i confocal microscope (Tokyo, Japan).

### 2.11. Statistical Analysis

The data are expressed as the mean ± SEM. Data were analyzed using one-way analysis of variance and Dunnett's multiple comparisons test. Results with a *P* value < 0.05 were considered to be statistically significant.

## 3. Results

### 3.1. HPLC Determination of the Nine Marker Components in MHT

**Figure 1:**
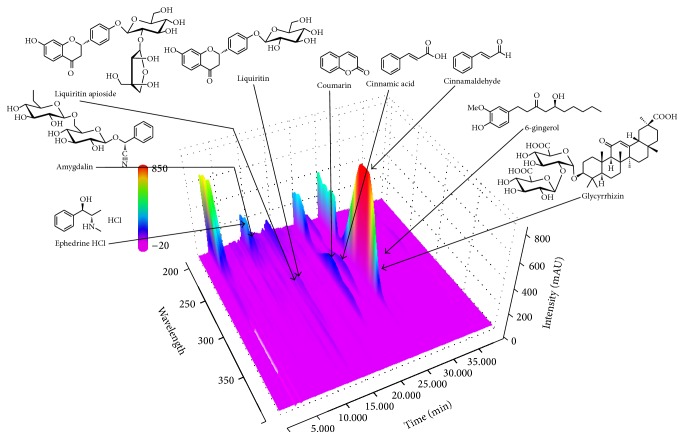
Three-dimensional chromatogram of MHT water extract by HPLC–PDA.

The optimized HPLC–PDA analytical method was successfully applied for the simultaneous determination of the 9 marker compounds in MHT extract. All compounds in MHT extract were identified based on the retention time and UV spectra of each reference standard. As a result, the 9 marker compounds including ephedrine HCl, amygdalin, liquiritin apioside, liquiritin, coumarin, cinnamic acid, cinnamaldehyde, glycyrrhizin, and 6-gingerol were detected at 9.09, 9.98, 13.89, 14.29, 20.34, 22.80, 25.49, 26.20, and 31.18 min, respectively (Figure 1). The concentrations of ephedrine HCl, amygdalin, liquiritin apioside, liquiritin, coumarin, cinnamic acid, cinnamaldehyde, glycyrrhizin, and 6-gingerol in MHT extract by the optimized analytical assay were 13.80, 21.57, 4.52, 2.08, 4.61, 1.75, 12.52, 6.71, and 0.19 mg/g, respectively.

### 3.2. Effects of MHT on the Cell Viability in HaCaT Keratinocytes

**Figure 2:**
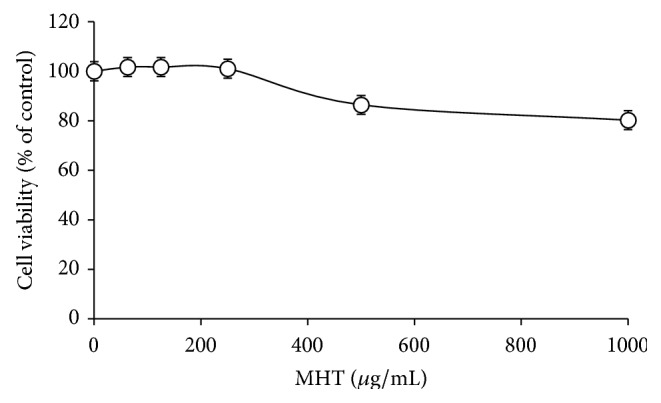
Cytotoxic effects of MHT in HaCaT cells. Cells were seeded onto 96-well plates and treated with various concentrations of MHT (0, 62.5, 125, 250, 500, or 1000 *μ*g/mL) for 24 h. Cell viability was assessed using CCK-8 assay. The values are expressed as the mean ± SEM of three independent experiments.

To obtain a suitable concentration range for investigating the effects of MHT on the viability in HaCaT cells (Figure 2), we treated cells with the concentration ranging from 62.5 to 1000 *μ*g/mL of MHT for 24 h. We observed no significant alteration in the cell viability following MHT treatment up to 1000 *μ*g/mL. Therefore, nontoxic concentrations (125, 250, or 500 *μ*g/mL) of MHT were used in subsequent experiments. Additionally, MHT had no effect on cell death by pH level (Supplementary Figure  1 in Supplementary Material available online at http://dx.doi.org/10.1155/2016/7831291).

### 3.3. Effects of MHT on the Inflammatory Chemokines in HaCaT Keratinocytes

**Figure 3:**
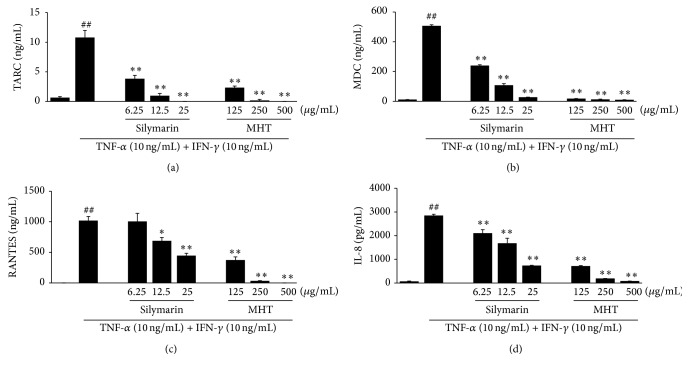
MHT inhibits production of inflammatory chemokines in TNF-*α* and IFN-*γ*-stimulated HaCaT cells. Production of TARC (a), MDC (b), RANTES (c), and IL-8 (d) were measured using the culture supernatants from cells treated with MHT (125, 250, or 500 *μ*g/mL) and TNF-*α* and IFN-*γ* (each 10 ng/mL, TI) for 24 h. Silymarin (6.25, 12.5, or 25 *μ*g/mL) was used as positive control. Values were expressed as mean ± SEM of three independent experiments. ^##^ *P* < 0.01 versus vehicle control cells; ^*∗*, *∗∗*^ *P* < 0.01 versus TI-treated cells.

**Figure 4:**
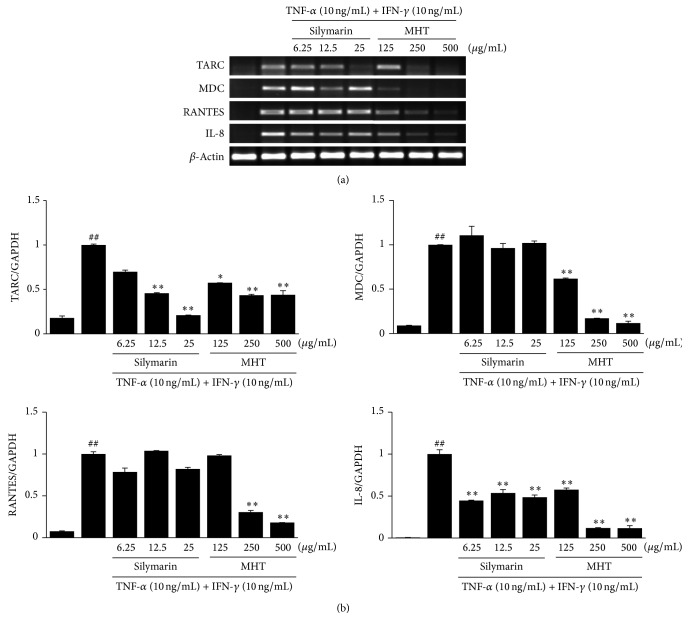
MHT inhibits the mRNA expression of inflammatory chemokines in TNF-*α* and IFN-*γ*-stimulated HaCaT cells. (a) RT-PCR was performed to determine the mRNA expression levels of TARC, MDC, RANTES, and IL-8. (b) The intensities of the PCR bands. Values were expressed as mean ± SEM of three independent experiments. ^##^ *P* < 0.01 versus vehicle control cells; ^*∗*, *∗∗*^ *P* < 0.01 versus TI-treated cells.

To assess the inhibitory effects of MHT, we cotreated HaCaT cells with TI in the absence or presence of MHT for 24 h, and the production of TARC, MDC, RANTES, and IL-8 was analyzed using ELISA. HaCaT cells treated with TI increased the chemokine production compared with the vehicle-treated cells. By contrast, MHT (125, 250, or 500 *μ*g/mL) significantly suppressed TI-stimulated production of TARC, MDC, RANTES, and IL-8 in a dose-dependent manner (*P* < 0.01), respectively (Figure 3). A positive control silymarin also suppressed the TI-stimulated chemokine production in a dose-dependent manner. We next analyzed the effects of MHT on chemokine mRNA expression in HaCaT cells costimulated with TI. Consistent with the results of ELISAs, stimulation with TI significantly increased the expression of TARC, MDC, RANTES, and IL-8 mRNA in HaCaT cells. Treatment of the cells with MHT significantly reduced the expression of TARC, MDC, RNATES, and IL-8 mRNA compared with TI in a dose-dependent manner (Figure 4). These results demonstrate that MHT can modulate production and expression of TI-stimulated Th2 inflammatory chemokine in HaCaT keratinocytes.

### 3.4. Effects of MHT on TNF-*α* and IFN-*γ*-Activated STAT1 Phosphorylation in HaCaT Keratinocytes

**Figure 5:**
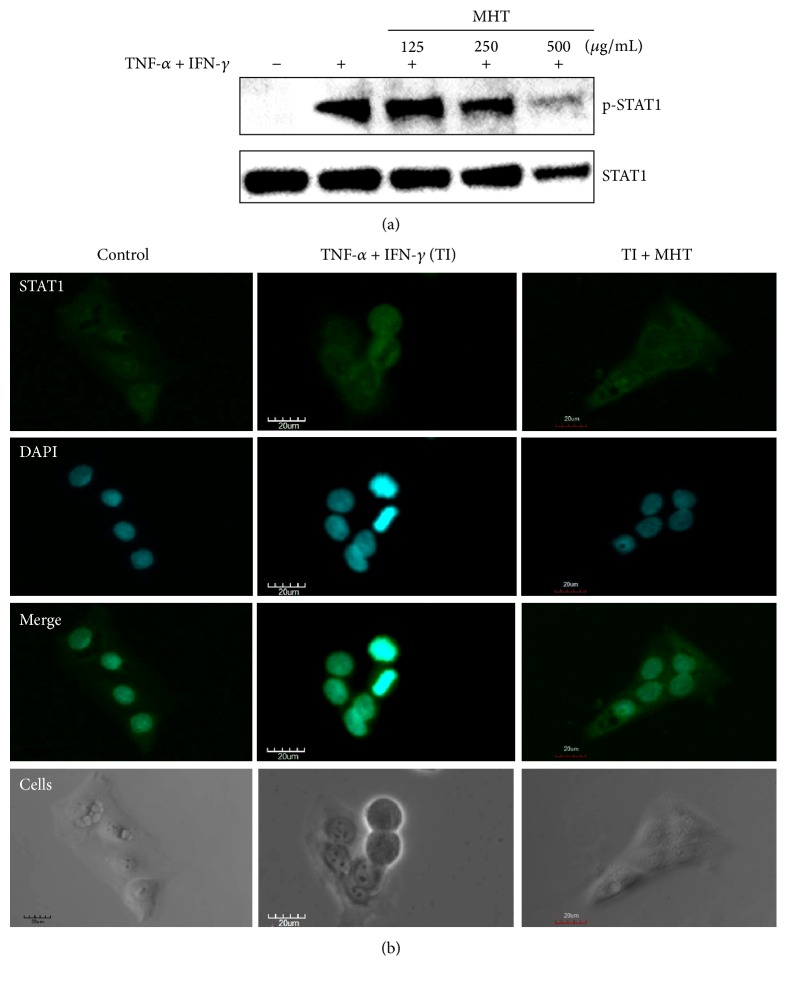
MHT suppresses STAT1 activation in TNF-*α* and IFN-*γ*-stimulated HaCaT cells. (a) Phosphorylation of STAT1 was measured using cells cotreated with MHT (125, 250, or 500 *μ*g/mL) and TNF-*α* and IFN-*γ* (each 10 ng/mL, TI) for 30 min by western blotting. (b) Cellular localization of STAT1 was analyzed by immunofluorescence staining. Cells were cotreated with MHT (500 *μ*g/mL) and TI for 30 min on glass coverslips and incubated with anti-STAT1 and FITC-conjugated secondary antibodies. The cells were fixed in 4% (v/v) methanol-free formaldehyde solution (pH 7.4), stained with anti-STAT1 (green). The stained cells were mounted in medium containing DAPI (blue) and visualized under an Olympus FLUOVIEW FV 10i confocal microscope.

We investigated the effect of MHT on phosphorylation of STAT1 in keratinocytes. HaCaT cells were pretreated with MHT for 1 h and then stimulated with TI for 30 min. MHT suppressed the TI-stimulated phosphorylation of STAT1 (Figure 5(a)). We also confirmed the effect of MHT on nuclear translocation of STAT1 by immunofluorescence analysis. STAT1 protein at cytosol was translocated to nucleus after TI stimulation whereas it was inhibited by MHT treatment (Figure 5(b)). These results indicate that MHT inhibits TI-stimulated STAT1 activation in HaCaT keratinocytes.

## 4. Discussion

MHT, which consists of six herbal medicines, is one of the traditional Korean medicine prescriptions and has been used for the treatment of headache, fever, and arthralgia which result from common cold in Korea [10]. The main ingredients of the MHT are known as follows: alkaloids (e.g., ephedrine and pseudoephedrine) from Ephedrae Herba [11], coumarin (e.g., coumarin, cinnamic acid, and cinnamaldehyde) from Cinnamomi Ramulus [12, 13], triterpene saponins (e.g., glycyrrhizin) and flavonoids (e.g., liquiritin and liquiritigenin) from Glycyrrhizae Radix et Rhizoma [14], cyanoglucosides (e.g., amygdalin) from Armeniacae Semen [11], phenols (e.g., 6-gingerol and 6-shogaol) from Zingiberis Rhizoma Crudus [15], and phenylpropanoids (e.g., ferulic acid) and flavonoids (e.g., kaempferol) from Allii Radix [16]. Among the various ingredients, we try simultaneous determination of the nine components ephedrine HCl, amygdalin, liquiritin apioside, liquiritin, coumarin, cinnamic acid, cinnamaldehyde, glycyrrhizin, and 6-gingerol in MHT water extract by a HPLC–PDA method. Consequently, amygdalin (21.57 mg/g), ephedrine HCl (13.80 mg/g), and cinnamaldehyde (12.52 mg/g), marker compounds of Armeniacae Semen, Ephedrae Herba, and Cinnamomi Ramulus, respectively, were detected as major compounds.

Among the major compounds of MHT, amygdalin, ephedrine HCl, and cinnamaldehyde have been reported to exhibit the effects of inflammatory responses through attenuation of iNOS, COX-2 expression via nuclear factor-kappa B signaling pathway, and PI3K/Akt/GSK3*β* pathway [17–19]. However, the effects of MHT on skin inflammatory diseases have not been reported. In the present study, we found that MHT inhibited production and mRNA expression of inflammatory chemokines such as TARC, MDC, RANTES, and IL-8 by suppressing STAT1 activation in TI-stimulated keratinocytes.

Chemokine products play important roles in the development of inflammatory cells in the skin. The secretion of these inflammatory chemokines is the first recruitment of inflammatory skin diseases and can be observed in immune cells, including lymphocytes, keratinocytes, and mast cells, which are activated by various stimuli [20]. Keratinocytes may promote an amplification on the inflammatory response with the production of TNF-*α* and IFN-*γ*. Stimulated keratinocytes have been reported as important sources of proinflammatory chemokines, including TARC, MDC, RNATES, and IL-8, which affect T lymphocyte differentiation and the recruitment of leukocytes to skin inflammatory diseases such as AD [21, 22]. Consistent with the results of previous studies, we found that stimulation with TI markedly increased the production and mRNA expression of TARC, MDC, RNATES, and IL-8 in HaCaT keratinocytes. By contrast, MHT treatment decreased both the production and mRNA expression of these chemokines compared with the TI-stimulated cells. These results indicate that MHT may have the inhibitory activity against skin inflammation through regulation of chemokines expression in keratinocytes.

STAT1 is a crucial molecule for the IFN-*γ*/cytokine signaling pathways [22]. These activated pathways can modulate the secretion of inflammatory mediators including TARC, MDC, and RANTES. Inhibition of STAT1 phosphorylation is considered to be an important step in treating skin inflammatory diseases [23]. To confirm the action mechanisms of the effect of MHT on the STAT1 activated by TI-stimulated HaCaT keratinocytes, we performed immunoblotting and immunocytochemistry assays (using anti-STAT1 antibody). In our study, MHT inhibited TI-stimulated STAT1 phosphorylation and nuclear translocation in HaCaT cells. These results suggest that MHT might block the induction inflammatory chemokines production by inhibiting STAT1 phosphorylation in TI-stimulated keratinocytes.

In summary, our present study demonstrates that MHT inhibited the production and mRNA expression of inflammatory chemokines including TARC, MDC, RANTES, and IL-8 by suppressing the activation of STAT1 in TI-stimulated HaCaT keratinocytes. Further investigation is required to elucidate the detailed mechanisms involved in anti-skin inflammatory diseases by MHT and to further confirm the inhibitory effect of MHT in skin diseases using an* in vivo* experimental model.

## Supplementary Material

Effects of pH level on MHT in TNF-α and IFN-γ-stimulated HaCaT cells. Cells were seeded onto 6-well plates and treated with various concentrations of MHT (125, 250, or 500 μg/mL) with TNF-α and IFN-γ (each 10 ng/mL) for 24 h. Celture supernatant was collected and pH values were measured. The values are expressed as the mean ± SEM of three independent experiments.

## Figures and Tables

**Table 1:** Composition of MHT.

Herbal medicine	Scientific name	Family	Origin	Ratio (%)
Ephedrae Herba	*Ephedra sinica *Stapf	Ephedraceae	China	34.9
Cinnamomi Ramulus	*Cinnamomum cassia *Presl	Lauraceae	Vietnam	23.3
Glycyrrhizae Radix et Rhizoma	*Glycyrrhiza uralensis *Fischer	Leguminosae	China	7.0
Armeniacae Semen	*Prunus armeniaca *Linne var. ansu Maximowicz	Rosaceae	China	11.6
Zingiberis Rhizoma Crudus	*Zingiber officinale* Roscoe	Zingiberaceae	Ulsan, Korea	11.6
Allii Radix	*Allium fistulosum* Linne	Liliaceae	Hanam, Korea	11.6

Total amount				100.0

**Table 2:** List of primer sequences for RT-qPCR.

Gene	Sequences of primers (5′ to 3′)		Size (bp)
	Forward	Reverse	
TARC	ACT GCT CCA GGG ATG CCA TCG TTT TT	ACA AGG GGA TGG GAT CTC CCT CAC TG	270
MDC	AGG ACA GAG CAT GGC TCG CCT ACA GA	TAA TGG CAG GGA GCT AGG GCT CCT GA	362
RANTES	CCC CGT GCC GAG ATC AAG GAG TAT TT	CGT CCA GCC TGG GGA AGG TTT TTG TA	313
IL-8	GTG GCT CTC TTG GCA GCC TTC CTG AT	TCT CCA CAA CCC TCT GCA CCC AGT TT	253
GAPDH	GTG ATG GCA TGG ACT GTG GT	AAG GGT CAT CAT CTC TGC CC	204
