# Supplementary material for: Ma Huang Tang Suppresses the Production and Expression of Inflammatory Chemokines via Downregulating STAT1 Phosphorylation in HaCaT Keratinocytes
Source: Evid Based Complement Alternat Med. 2016 Oct 26;2016:7831291. doi: 10.1155/2016/7831291 (PMC5101387; doi:10.1155/2016/7831291)
Supplement: Supplementary file 1 — Effects of pH level on MHT in TNF-α and IFN-γ-stimulated HaCaT cells. Cells were seeded onto 6-well plates and treated with various concentrations of MHT (125, 250, or 500 μg/mL) with TNF-α and IFN-γ (each 10 ng/mL) for 24 h. Celture supernatant was collected and pH values were measured. The values are expressed as the mean ± SEM of three independent experiments. [file 7831291.f1.pdf]

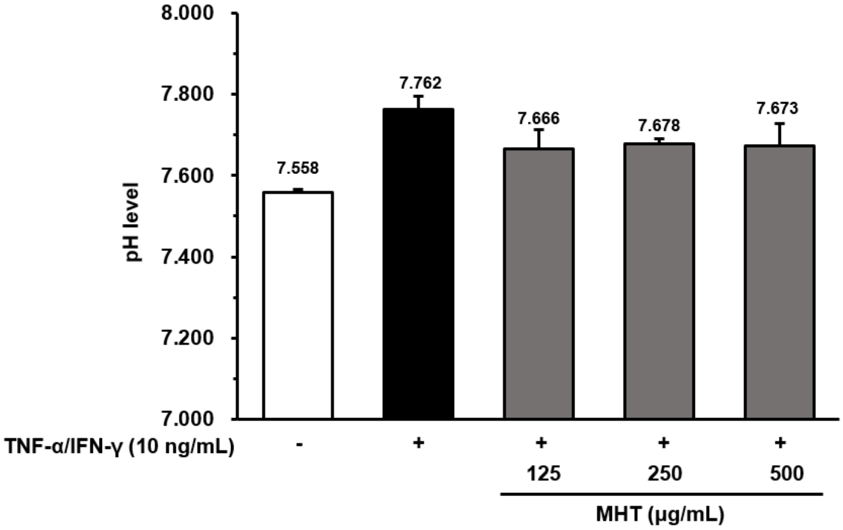

Supplementary Figure 1. Effects of pH level on MHT in TNF-α and IFN-γ-stimulated HaCaT cells. Cells were seeded onto 6-well plates and treated with various concentrations of MHT (125, 250, or 500 μg/mL) with TNF-α and IFN-γ (each 10 ng/mL) for 24 h. The values are expressed as the mean ± SEM of three independent experiments.
